# Prevalence of Past-Year Dental Visit Among US Adults Aged 50 Years or Older, With Selected Chronic Diseases, 2018

**DOI:** 10.5888/pcd18.200576

**Published:** 2021-04-29

**Authors:** Nita Patel, Rebecca Fils-Aime, Chien-Hsun Li, Mei Lin, Valerie Robison

**Affiliations:** 1 Centers for Disease Control and Prevention, Division of Oral Health, Atlanta, Georgia; 2 Now with Emory University, Atlanta, Georgia; 3 CyberData Technologies Inc, Herndon, Virginia

## Abstract

In this study, we used data from the Behavioral Risk Factor Surveillance System to conduct multivariable analyses to examine whether having selected chronic diseases was associated with lower past-year dental service utilization among US adults aged 50 years or older. We found consistent lower dental service utilization among older adults with diabetes, heart disease or stroke, and chronic obstructive pulmonary disease (COPD) compared with those without the disease after adjusting for sociodemographic characteristics. We also found lower dental service utilization among older adults with lower income, less education, and no health care coverage and among those who smoked. Effective interventions are needed to reduce disparities in access to dental care among at-risk and vulnerable populations.

**Summary**

**What is already known on this topic?**

Although routine dental care is an important component of maintaining overall health, little is known about dental service utilization among adults with chronic conditions.

**What is added by this report?**

We found small but consistent lower dental service utilization among older adults with diabetes, heart disease or stroke, and chronic obstructive pulmonary disease compared with those without the disease after adjusting for sociodemographic characteristics. We also found lower dental service utilization among older adults who smoked and who had lower income, less education, and no health care coverage.

**What are the implications for public health practice?**

Our findings suggest the need to examine effective interventions to increase dental service utilization among at-risk and vulnerable populations.

## Objective

Adults who report chronic conditions have a higher prevalence of unmet dental treatment needs (1) and resulting tooth loss (2) than their counterparts without chronic conditions; adults reporting diabetes, heart disease, or rheumatoid arthritis are at least 50% more likely to have severe tooth loss that results in difficulty eating healthy foods. Tentative evidence suggests that the level of the periodontal pathogens and inflammatory response from periodontal disease could increase the risk of systemic diseases such as cardiovascular disease (3). A Cochrane review (4) found evidence that treating periodontitis improved glycemic control among people with diabetes. Although routine dental care is an important component of maintaining overall health, little is known about dental service utilization among adults with chronic conditions.

## Methods

We used data from the 2018 Behavioral Risk Factor Surveillance System (BRFSS), a cross-sectional, annual landline and cellular telephone survey of noninstitutionalized adults aged 18 years or older living in the 50 US states, the District of Columbia, and US territories on health-related risk behaviors, chronic health conditions, and use of preventive services (5). Individuals without teeth (ie, edentate) are less likely to see the dentist (6); we restricted our study population to respondents who had at least 1 permanent tooth and were aged 50 years or older (N = 242,452).

The outcome variable, dental service utilization, was defined as visiting a dentist or dental clinic for any reason within the past year. We included chronic diseases associated with periodontal disease: arthritis, diabetes, heart diseases, and chronic obstructive pulmonary disease (COPD). We categorized heart attack, stroke, and coronary heart disease as heart disease. We assessed smoking status, a well-established risk factor for periodontal disease (7). Current cigarette smoking was defined as answering yes to the question, “Have you smoked at least 100 cigarettes in your entire life?” and answering “every day” or “some days” to the question, “Do you now smoke cigarettes every day, some days, or not at all?”

Analysis was conducted using SAS-Callable SUDAAN (SAS Institute, Inc) to account for the complex sampling design. For bivariate analyses, we used χ^2^ tests to calculate prevalence of past-year dental visit by chronic conditions, regions, and selected sociodemographic characteristics. We used *t *tests for the multivariable analysis to determine adjusted prevalence ratios (aPRs) and 95% CIs for having a past-year dental visit by chronic conditions, adjusting for age, sex, race/ethnicity, annual household income, education, health care coverage, cigarette smoking status, and region. Significance was set at *P* < .001.

## Results

Approximately 7 in 10 dentate adults aged 50 years or older reported having a dental visit in the past year (72.4%) (Table 1). Crude prevalence of past-year dental visit was lower among adults who were male; were aged 50 to 64 years; were non-Hispanic black, Hispanic, or non-Hispanic other; had less than a college or technical school education; had an annual household income less than $75,000; had no health care coverage; and resided in regions other than the Northeast (Table 2). Crude prevalence of having a past-year dental visit was lower among older adults with arthritis; diabetes; heart attack, stroke, or coronary heart disease; and COPD. Current and former smokers were also less likely to have a past year dental visit than nonsmokers (Table 2).

**Table 1:** Percentage of Dentate^a^ Adults Aged ≥50 Years, by Selected Characteristics, Behavioral Risk Factor Surveillance System, United States, 2018

Characteristic	No. of Respondents (N = 242,452)	Weighted Percentage (SE)
**Demographic Characteristics**		
**Age, y**		
50–64	115,367	57.6 (0.2)
≥65	127,085	42.4 (0.2)
**Sex**		
Male	105,672	46.9 (0.2)
Female	136,239	53.1 (0.2)
**Race/ethnicity**		
Non-Hispanic White	197,581	73.4 (0.3)
Non-Hispanic Black	17,628	9.9 (0.1)
Hispanic	10,229	10.6 (0.2)
Non-Hispanic other	12,305	6.1 (0.2)
**Education**		
Did not graduate from high school	13,679	11.8 (0.2)
High school graduate	63,005	26.7 (0.2)
Attended college or technical school	66,414	31.4 (0.2)
College or technical school graduate	98,577	30.2 (0.2)
**Annual household income, $**		
≤24,999	44,697	23.2 (0.2)
25,000–49,999	49,689	23.2 (0.2)
50,000–74,999	34,540	16.1 (0.2)
≥75,000	70,203	37.4 (0.2)
**Health care coverage**		
Yes	230,679	93.6 (0.1)
No	11,129	6.4 (0.1)
**US region** b		
Northeast	78,648	24.1 (0.2)
Southeast	35,943	20.3 (0.1)
Southwest	18,490	11.3 (0.2)
Midwest	55,579	20.8 (0.1)
West	53,792	23.4 (0.2)
**Health Status and Chronic Diseases**		
**Arthritis**		
Yes	107,730	41.6 (0.2)
No	133,430	58.4 (0.2)
**Diabetes**		
Yes	42,415	18.3 (0.2)
No	199,705	81.7 (0.2)
**Heart attack, stroke, or coronary heart disease**		
Yes	36,703	14.3 (0.2)
No	205,653	85.7 (0.2)
**Chronic obstructive pulmonary disease**		
Yes	22,762	9.3 (0.1)
No	218,478	90.7 (0.1)
**History of cigarette smoking**		
Current smoker	25,509	12.0 (0.2)
Former smoker	77,314	32.1 (0.2)
Never smoked	131,447	55.9 (0.2)
**Past-year dental visit**		
Yes	180,015	72.4 (0.2)
No	60,612	27.6 (0.2)

a Dentate is defined as individuals with at least 1 permanent tooth.

b For our analysis, the Northeast region includes HHS Regions 1 (Maine, Massachusetts, New Hampshire, Rhode Island, and Vermont), 2 (New Jersey, New York), and 3 (District of Columbia, Maryland, Pennsylvania, Virginia, and West Virginia); the Southeast region includes HHS Region 4 (Alabama, Florida, Georgia, Kentucky, Mississippi, North Carolina, South Carolina, and Tennessee); the Southwest region includes HHS Region 6 (Arkansas, Louisiana, New Mexico, Oklahoma and Texas); the Midwest region includes HHS Regions 5 (Indiana, Illinois, Michigan, Minnesota, Ohio, and Wisconsin) and 7 (Iowa, Kansas, Missouri, and Nebraska); and the West region includes HHS Regions 8 (Colorado, Montana, North Dakota, South Dakota, Utah and Wyoming), 9 (Arizona, California, Hawaii, Nevada), and 10 (Alaska, Idaho, Oregon, and Washington).

**Table 2:** Crude Prevalence and Adjusted Prevalence Ratio of Past-Year Dental Visit Among Dentate^a^ Adults Aged ≥50 Years, Behavioral Risk Factor Surveillance System, United States, 2018

Characteristic	Crude Prevalence of Past-Year Dental Visit (95% CI)	*P* Value^b^	aPR (95% CI)	*P* Value^c^
**Overall**	72.4 (72.0–72.8)	NA	NA	NA
**Sex**				
Male	69.9 (69.2–70.5)	<.001	1 [Reference]	
Female	74.7 (74.1–75.2)		1.08 (1.07–1.10)	<.001
**Age, y**				
50–64	70.8 (70.2–71.3)	<.001	1 [Reference]	
≥65	74.6 (74.0–75.3)		1.06 (1.05–1.08)	<.001
**Race/ethnicity**				
Non-Hispanic White	76.0 (75.6–76.4)	<.001	1 [Reference]	
Non-Hispanic Black	60.3 (58.7–61.8)		0.92 (0.89–0.94)	<.001
Hispanic	60.3 (58.2–62.4)		1.00 (0.97–1.04)	0.82
Non-Hispanic other	70.1 (67.4–72.7)		0.97 (0.93–1.01)	0.09
**Education**				
Did not graduate from high school	48.7 (46.8–50.7)	<.001	0.77 (0.74–0.81)	<.001
High school graduate	66.7 (65.9–67.5)		0.91 (0.89–0.92)	<.001
Attended some college or technical school	73.9 (73.1–74.6)		0.95 (0.94–0.97)	<.001
College or technical school graduate	85.1 (84.6–85.6)		1 [Reference]	
**Annual household income, $**				
≤24,999	51.6 (50.5–52.7)	<.001	0.70 (0.68–0.72)	<.001
25,000–49,999	67.0 (66.0–68.1)		0.83 (0.82–0.85)	<.001
50,000–74,999	77.8 (76.7–78.8)		0.93 (0.92–0.95)	<.001
≥75,000	86.2 (85.6–86.7)		1 [Reference]	
**Health care coverage**				
Yes	74.3 (73.9–74.7)	<.001	1 [Reference]	
No	44.9 (42.7–47.2)		0.75 (0.70–0.79)	<.001
**Arthritis**				
Yes	71.4 (70.7–72.0)	<.001	1.01 (1.00–1.02)	.16
No	73.2 (72.6–73.8)		1 [Reference]	
**Diabetes**				
Yes	64.7 (63.6–65.9)	<.001	0.95 (0.93–0.97)	<.001
No	74.1 (73.7–74.6)		1 [Reference]	
**Heart attack, stroke, or coronary heart disease**				
Yes	63.9 (62.7–65.0)	<.001	0.95 (0.92–0.97)	<.001
No	73.8 (73.4–74.3)		1 [Reference]	
**COPD**				
Yes	59.3 (57.7–60.8)	<.001	0.93 (0.91–0.96)	<.001
No	73.8 (73.4–74.3)		1 [Reference]	
**History of cigarette smoking**				
Current smoker	53.3 (51.9–54.8)	<.001	0.82 (0.79–0.85)	<.001
Former smoker	73.0 (72.3–73.7)		0.98 (0.97–0.99)	0.001
Never smoked	76.3 (75.7–76.8)		1 [Reference]	
**US region** d				
Northeast	76.2 (75.4–76.8)	<.001	1 [Reference]	
Southeast	69.4 (68.5–70.3)		0.97 (0.95–0.98)	<.001
Southwest	64.7 (62.7–66.7)		0.91 (0.88–0.94)	< .001
Midwest	74.3 (73.6–74.9)		0.99 (0.98–1.01)	.33
West	73.2 (72.2–74.1)		0.97 (0.96–0.99)	.001

Abbreviations: aPR, adjusted prevalence ratio; COPD, chronic obstructive pulmonary disease; NA, not applicable.

a Dentate is defined as individuals with at least 1 permanent tooth.

b Chi-square test was used for the bivariate analysis.

c *t* test was used for the adjusted prevalence ratio analysis.

d For our analysis, the Northeast region includes HHS Regions 1 (Maine, Massachusetts, New Hampshire, Rhode Island, and Vermont), 2 (New Jersey, New York), and 3 (District of Columbia, Maryland, Pennsylvania, Virginia, and West Virginia); the Southeast region includes HHS Region 4 (Alabama, Florida, Georgia, Kentucky, Mississippi, North Carolina, South Carolina, and Tennessee); the Southwest region includes HHS Region 6 (Arkansas, Louisiana, New Mexico, Oklahoma and Texas); the Midwest region includes HHS Regions 5 (Indiana, Illinois, Michigan, Minnesota, Ohio, and Wisconsin) and 7 (Iowa, Kansas, Missouri, and Nebraska); and the West region includes HHS Regions 8 (Colorado, Montana, North Dakota, South Dakota, Utah and Wyoming), 9 (Arizona, California, Hawaii, Nevada), and 10 (Alaska, Idaho, Oregon, and Washington).

In the adjusted model, older adults with diabetes; heart attack, stroke, or coronary heart disease; or COPD consistently had significantly lower dental service utilization compared with those without the disease, although the magnitude of the associations was small (Table 2). In addition, the associations between past-year dental visit and education, income, health insurance coverage, and cigarette smoking status remained pronounced in the adjusted model. Having a past-year dental visit was lower among older adults who did not graduate from high school (aPR, 0.77; 95% CI, 0.74–0.81), who had an annual household income <$24,999 (aPR, 0.70; 95% CI, 0.68–0.72), or were without health care coverage (aPR, 0.75; 95% CI, 0.70–0.79) than older adults with a college degree or higher education, with an annual household income of $75,000 or more, or with health care coverage. Current smokers had lower dental service utilization than never smokers (aPR, 0.82; 95% CI, 0.79–0.85).

## Discussion

To our knowledge, our study is the first to describe dental service utilization among older adults with selected chronic diseases. Although the magnitude of the association was small, we found consistently lower dental service utilization among older adults with diabetes, heart disease or stroke, and COPD, even after adjusting for sociodemographic characteristics. Similar to other studies (8,9), we found that disparities in dental service utilization among older adults continue to persist by education, income, and health insurance status, as well as among cigarette smokers.

Lower dental service utilization among older adults could be, in part, due to loss of employer-based health insurance after retirement. Not all adults who have health insurance have dental insurance. Original Medicare does not provide routine dental services. Medicare Advantage Plans may cover dental services, but level of dental services and out-of-pocket costs vary by plan. We found that having chronic conditions was a barrier to dental service utilization. Individuals with systemic or chronic diseases are likely to prioritize their medical needs (9) over dental needs. Also, poor health associated with some chronic diseases could limit mobility among the elderly and subsequently affect access to dental services (9).

In 2011, the Institute of Medicine proposed integrating oral health with the medical health care system to promote better health and improve access to both dental and medical preventive services (10). Our findings suggest the need to have continued national dialogue to foster interprofessional and interprogram collaboration, examine how oral health care and medical care intersect, and identify opportunities where the 2 disparate health systems can potentially integrate to facilitate better care coordination for older adults with chronic diseases. Additional strategies may include systematically reviewing examples of successful models of medical–dental integration to identify best practices that increase dental service utilization among older adults with chronic diseases. Some short-term strategies may include educating health care providers about the higher need for routine dental care among older adults with chronic diseases.

Our study had several limitations. First, BRFSS data are self-reported and subject to both recall and social desirability bias. Second, BRFSS does not assess dental insurance coverage. Our use of health care coverage as a proxy indicator for dental insurance may have resulted in underestimation of dental service utilization.

In conclusion, effective interventions are needed to reduce disparities in access to dental care among at-risk and vulnerable populations.
